# Cranio-osteoarthropathy associated with inflammatory arthritis

**DOI:** 10.1186/1546-0096-12-S1-P209

**Published:** 2014-09-17

**Authors:** Sunil Sampath, Kirsty Fletton, Clare Pain, Liza McCann, Gavin Cleary, Michael Beresford, Eileen Baildam

**Affiliations:** 1 Paediatric Rheumatology, Alder Hey Children’s NHS foundation trust, Liverpool, UK; 2 Medical school, University of Liverpool, Liverpool, UK; 3 Department of Women’s and Children’s Health, Institute of Translational Medicine, Liverpool, UK

## Introduction

Cranio-Osteoarthropathy (COA) is a very rare form of primary hypertrophic osteoarthropathy (HOA). HOA is characterised by digital clubbing, arthropathy, pachyderma and sub-periosteal new bone formation along shafts of long bones. In COA, decreased neurocranium ossification is seen without the pachyderma feature.

## Objectives

Although joint effusions are described in COA, to the best of our knowledge, it has not been associated with inflammatory arthritis. Here we describe a female with COA and inflammatory arthritis.

## Methods

Describe the evolution of the clinical features in a child with COA and review of literature.

## Results

### Case report

Female child born at term to consanguineous parents from Asian ethnicity needed surgical ligation of a large patent ductus arteriosus (PDA) during the neonatal period. Finger clubbing was noted first from 6 weeks of age; over the next few years, despite detailed assessment, no secondary cause could be found. At the age of 7 years, she presented to the rheumatology team with swelling, pain and stiffness in her knees and ankles; although her joint symptoms dated back to since was 3 years old. She always had excessively sweaty hands and feet. On examination she had marked clubbing of her fingers and toes, with synovial effusions and thickening of knees and ankles. She also had long fingers and toes and prominent nose. Fingers were particularly hypermobile and she had prominent shoulders.

Her initial synovial fluid analyses were consistent with primary inflammatory arthropathy, although subsequent synovial analysis has been of ‘non-inflammatory type’. Over the next few years she received multiple intra-articular joint steroid injections, with short lasting symptom relief.

Plain radiographs from childhood had evidence of wormian bones. Radiographs also revealed periosteal reaction in all distal phalanges and femurs. Gadolinium enhanced MRI was consistent with inflammatory arthritis. Auto-antibody screen has always been negative, persistently raised IgG and persistent mild elevation of angiotensin converting enzyme (ACE) levels.Her previous treatments for inflammatory joint disease have included methotrexate, etanercept and rituximab.She is seeing ophthalmologists for meiobian gland dysfunction, corneal neovascularisation and punctate epithelial erosion. She is waiting to see psychology for low mood.

## Conclusion

Primary HOA is a genetic disorder involved mutation in HPGD gene that encodes 15-hydroxyprostaglandin dehydrogenase, which is the primary enzyme response for prostaglandin degradation, increased incidence of PDA in HOA is thus not surprising. Several treatments including NSAIDs, colchicine and pamidronate are proposed for Joint symptoms in COA. Pachydermoperiostosis, another form of HOA has a self-limiting course, an active adolescent phase followed by a quiescent adult phase; however long term prognosis of COA is not well described in literature.

## Conclusion

Here, we have describe the features and treatment response in a case of COA.Inflammatory arthritis is not previously well described in COA and managing joint symptoms can be challenging.

## Disclosure of interest

None declared.

